# Comparison of post-COVID-19 symptoms in patients infected with the SARS-CoV-2 variants delta and omicron—results of the Cross-Sectoral Platform of the German National Pandemic Cohort Network (NAPKON-SUEP)

**DOI:** 10.1007/s15010-024-02270-5

**Published:** 2024-05-03

**Authors:** Sina M. Pütz, Katharina S. Appel, Olga Miljukov, Johannes Schneider, Marylyn M. Addo, Robert Bals, Sven Bercker, Sabine Blaschke, Isabel Bröhl, Nikolaus Büchner, Hiwa Dashti, Johanna Erber, Anette Friedrichs, Ramsia Geisler, Siri Göpel, Marina Hagen, Frank Hanses, Björn-Erik Ole Jensen, Maria Keul, Adalbert Krawczyk, Bettina Lorenz-Depiereux, Patrick Meybohm, Milena Milovanovic, Lazar Mitrov, Carolin Nürnberger, Wilfried Obst, Christoph Römmele, Christian Schäfer, Christian Scheer, Margarete Scherer, Julia Schmidt, Kristina Seibel, Shimita Sikdar, Johannes Josef Tebbe, Phil-Robin Tepasse, Philipp Thelen, Maria J. G. T. Vehreschild, Christina Weismantel, J. Janne Vehreschild

**Affiliations:** 1 Faculty of Medicine, Department I of Internal Medicine, Center for Integrated Oncology Aachen Bonn Cologne Duesseldorf, University of Cologne, University Hospital Cologne, Weißhausstraße 24, 50939 Cologne, Germany; 2 Center for Internal Medicine, Medical Department 2 (Hematology/Oncology and Infectious Diseases), Goethe University Frankfurt, University Hospital, Frankfurt, Germany; 3 Faculty of Medicine, Department I of Internal Medicine, University of Cologne, University Hospital Cologne, Cologne, Germany; 4 Faculty of Medicine, Institute for Clinical Epidemiology and Biometry, University of Wuerzburg, Wuerzburg, Germany; 5 Institute for Medical Data Sciences, University Hospital Wuerzburg, Wuerzburg, Germany; 6 Institute for Infection Research and Vaccine Development (IIRVD), Center for Internal Medicine, University Medical Center Hamburg-Eppendorf, Hamburg, Germany; 7 1St Department of Medicine, Division of Infectious Diseases, Center for Internal Medicine, University Medical Center Hamburg-Eppendorf, Hamburg, Germany; 8 German Centre for Infection Research (DZIF), Partner Site Hamburg-Lübeck-Borstel-Riems, Hamburg, Germany; 9 Department of Internal Medicine V, Saarland University Medical Center, Homburg, Germany; 10 Helmholtz-Institute for Phamaceutic Research Saarland (HIPS), Saarbrücken, Germany; 11 Department of Anesthesiology and Intensive Care Medicine, University of Leipzig Medical Faculty, Leipzig, Germany; 12 Emergency Department, University Medical Center Goettingen, Goettingen, Germany; 13 Department of Respiratory and Sleep Medicine, Helios Hospital Duisburg, , Duisburg, Germany; 14 BAG Hausarztpraxis Dashti, Eberswalde, Germany; 15 TUM School of Medicine and Health, Department of Clinical Medicine, Clinical Department for Internal Medicine II, University Medical Center, Technical University of Munich, Munich, Germany; 16 Department of Internal Medicine I, University Hospital Schleswig-Holstein, Campus Kiel, Kiel, Germany; 17 Department of Internal Medicine 1, University Hospital Tübingen, Tübingen, Germany; 18 German Centre for Infection Research (DZIF) Clinical Research Unit for Healthcare Associated and Antibiotic Resistant Bacterial Infections, Tübingen, Germany; 19 Emergency Department and Department for Infection Control and Infectious Diseases, University Hospital Regensburg, Regensburg, Germany; 20 Department of Gastroenterology, Hepatology and Infectious Diseases, Medical Faculty, University Hospital Duesseldorf, Heinrich-Heine-University, Duesseldorf, Germany; 21 MVZ Altstadt-Carree Fulda GmbH, Fulda, Germany; 22 Department of Infectious Diseases, West German Centre of Infectious Diseases, University Hospital Essen, University of Duisburg-Essen, Essen, Germany; 23 Institute for Virology, University Hospital Essen, University Duisburg-Essen, Essen, Germany; 24 Institute of Epidemiology, Helmholtz Zentrum München, Munich, Germany; 25 Department of Anaesthesiology, Intensive Care, Emergency and Pain Medicine, University Hospital Würzburg, Würzburg, Germany; 26 Malteser Krankenhaus St. Franziskus Hospital Flensburg, Flensburg, Germany; 27 Department of Gastroenterology, Hepatology and Infectious Diseases, University Hospital Magdeburg, Medical Faculty of Otto-Von-Guericke University Magdeburg, Magdeburg, Germany; 28 Clinic for Internal Medicine III, Gastroenterology and Infectious Diseases, University Hospital of Augsburg, Stenglinstraße 2, 86156 Augsburg, Germany; 29 Institute of Clinical Chemistry and Laboratory Medicine, University Medicine Greifswald, Greifswald, Germany; 30 Department of Anesthesiology, Intensive Care Medicine, Emergency Medicine and Pain Medicine, University Medicine Greifswald, Greifswald, Germany; 31 Klinikum Lippe Department of Gastroenterology and Infectious Diseases, Bielefeld University, Medical School OWL, Bielefeld, Germany; 32 Department of Medicine B for Gastroenterology, Hepatology, Endocrinology and Clinical Infectiology, University Hospital Muenster, Münster, Germany; 33 Institute of Medical Microbiology and Virology, Carl Von Ossietzky University of Oldenburg, Oldenburg, Germany; 34 Department of Internal Medicine, Infectious Diseases, Goethe University, University Hospital Frankfurt, Frankfurt Am Main, Germany; 35 German Centre for Infection Research (DZIF), Partner Site Bonn-Cologne, Cologne, Germany

**Keywords:** Post-covid-19 condition, SARS-CoV-2 variants, Health-related quality of life, Multicenter prospective cohort study

## Abstract

**Purpose:**

The influence of new SARS-CoV-2 variants on the post-COVID-19 condition (PCC) remains unanswered. Therefore, we examined the prevalence and predictors of PCC-related symptoms in patients infected with the SARS-CoV-2 variants delta or omicron.

**Methods:**

We compared prevalences and risk factors of acute and PCC-related symptoms three months after primary infection (3MFU) between delta- and omicron-infected patients from the Cross-Sectoral Platform of the German National Pandemic Cohort Network. Health-related quality of life (HrQoL) was determined by the EQ-5D-5L index score and trend groups were calculated to describe changes of HrQoL between different time points.

**Results:**

We considered 758 patients for our analysis (delta: n = 341; omicron: n = 417). Compared with omicron patients, delta patients had a similar prevalence of PCC at the 3MFU (p = 0.354), whereby fatigue occurred most frequently (n = 256, 34%). HrQoL was comparable between the groups with the lowest EQ-5D-5L index score (0.75, 95% CI 0.73–0.78) at disease onset. While most patients (69%, n = 348) never showed a declined HrQoL, it deteriorated substantially in 37 patients (7%) from the acute phase to the 3MFU of which 27 were infected with omicron.

**Conclusion:**

With quality-controlled data from a multicenter cohort, we showed that PCC is an equally common challenge for patients infected with the SARS-CoV-2 variants delta and omicron at least for the German population. Developing the EQ-5D-5L index score trend groups showed that over two thirds of patients did not experience any restrictions in their HrQoL due to or after the SARS-CoV-2 infection at the 3MFU.

**Clinical Trail registration:**

The cohort is registered at ClinicalTrials.gov since February 24, 2021 (Identifier: NCT04768998).

**Supplementary Information:**

The online version contains supplementary material available at 10.1007/s15010-024-02270-5.

## Introduction

The emergence of new Severe Acute Respiratory Syndrome Coronavirus 2 (SARS-CoV-2) variants poses constant new challenges for clinicians and scientists, after the wild-type variant initially predominated. The first SARS-CoV-2 variant of concern—lineage B.1.1.7, named “alpha”—was identified in autumn 2020 in the United Kingdom [1]. The second SARS-CoV-2 variant of concern with a high impact in Europe was the lineage B.1.617.2, called “delta” variant. It was first identified in India in December 2020 and spread rapidly and globally [2, 3]. In mid-July 2021, more than 95% of SARS-CoV-2 infections in Germany were caused by the delta variant [4]. Since October 2021, the delta variant completely replaced the alpha variant in Germany [5]. In November 2021, a new SARS-CoV-2 variant appeared in South Africa: the lineage B.1.1.529 – named “omicron” variant [6]. Subvariants of the omicron variant are currently still predominant in Germany [7].

In the acute phase of Coronavirus Disease 2019 (COVID-19), both alpha and delta variants, were associated with a higher risk for hospitalization [8–10] and mortality [11–13] compared to the wild-type variant. In contrast, as the omicron variant spread, the number of hospital admissions decreased and the acute disease courses were mostly less severe [14–20] which was positively influenced by the improved immunity due to increased vaccination rates and previous infections. Acute symptoms like loss of smell and taste, sneezing, runny nose, and brain fog were less common in patients infected with the omicron variant (“omicron patients”) compared to the delta variant (“delta patients”) [16, 21]. In contrast, sore throat appeared more often in omicron than in delta patients.

After the acute phase of the COVID-19 disease, concerning amounts of patients develop post-COVID-19 condition (PCC) [22]. This usually appears within three months from the symptom onset of the primary SARS-CoV-2 infection or persists from the acute phase, lasts for at least two months, and cannot be explained by an alternative diagnosis, as defined by the World Health Organization (WHO) [23]. Frequent symptoms of PCC include fatigue, dyspnea, and cognitive impairment [24–26]. In addition, many other symptoms have been described, which can be grouped e.g. in cardiovascular, neurological, respiratory, and musculoskeletal categories [27]. Pain, including manifestations like chest pain [28] or headache [26], is also a frequently observed PCC-related symptom [29, 30].

While many studies have addressed the general occurrence of the PCC, comparative analyses between variants, especially the delta and omicron variant, are rare. Furthermore, extensive descriptions exist regarding the varied effects of different variants on the acute phase of COVID-19 disease. However, there are many open questions regarding the PCC depending on the SARS-CoV-2 variant.

In our study, we analyzed the prevalence and predictors of acute and PCC-related symptoms in patients infected with the delta or omicron variant. We investigated whether the presence of acute symptoms was associated with PCC under consideration of various co-factors three months after primary infection. In addition, we examined the change in the Health-related quality of life (HrQoL) in the course of the disease using the EQ-5D-5L, a validated questionnaire that assesses five dimensions of health.

## Methods

### Study procedures

#### Patient recruitment

For our analysis, data from the Cross-Sectoral Platform of the German National Pandemic Cohort Network (NAPKON-SUEP) was used, which contains in- and outpatients from German university hospitals as well as from non-university hospitals and the ambulant sector [31]. Patients were prospectively recruited within seven days after the day of positive SARS-CoV-2 detection, representing the baseline visit. During the acute phase of infection, weekly study visits (for in- and outpatients) and intervening documentation visits (only for inpatients) took place to collect data on patient status, vital and laboratory parameters. If infection-associated complications or clinical aggravation occurred, additional visits were conducted to assess severity and the current patient status. For inpatients, the end of hospitalization marked the end of acute phase. Here, a detailed study visit took place. In the outpatient setting, the end of acute phase was defined as 48 h without fever or no further aggravation or complications for symptomatic patients. If patients were asymptomatic, the end of acute phase visit took place five to nine days after baseline visit or if no new aggravation of the existing complications had occurred for seven days. In the NAPKON-SUEP, the follow-up visits took place three and 12 months after primary infection with additional telephone interviews every six weeks. For this analysis, we focused on the 3-months follow-up (3MFU). Patients were either examined and questioned in person in the study center (most of inpatient settings during acute phase) or questioned via phone call (all outpatient settings during acute phase, some inpatient settings during acute phase). Further details about the visit structure of the NAPKON-SUEP were described previously [32].

#### Recording of symptoms

In the acute phase, patients were directly asked for symptoms by the study personnel. For this analysis, the acute symptoms were divided into four groups: (1) general symptoms including fever, loss of appetite, lymphadenopathy, dizziness, headache, earache, chest pain, myalgia, arthralgia, skin or mucosal changes, apathy, and limb pain; (2) respiratory symptoms including sore throat, rhinorrhea, nasal congestion, sneezing, cough, dyspnea, and wheezing; (3) neurological symptoms including olfactory disorder, taste disorder, visual disorder, oculomotor disorders, aphasia, neuralgia, ataxia, confusion, cognitive impairment, and fatigue; and (4) gastrointestinal symptoms including abdominal pain, nausea, vomiting, and diarrhea. At the 3MFU, patients were asked three screening questions targeted to pain, dyspnea, and fatigue to assess common PCC-related symptoms. Thereby patients were asked for any sort of pain without further distinctions in the type or localization of the pain. The EQ-5D-5L and the EQ visual analogue scale (EQ VAS) were used to detect HrQoL by Patient Reported Outcome Measures (PROMs) and were recorded at baseline, at the end of the acute phase and at the 3MFU. The EQ-5D-5L index scores were calculated according to the German Value Set for the EQ-5D-5L with ranges between − 0.661 (extreme problems in all 5 dimensions) and 1 (no problems in any dimension) [33, 34].

Since attribution of symptoms to either PCC or other health conditions is unreliable in an epidemiological setting, we created groups of patients with different trends of EQ-5D-5L index scores. We hypothesized that patients with actual PCC would have a further decrease in HrQoL after recovery from acute symptoms of infection. To test our hypothesis, EQ-5D-5L index score trends between the baseline and the end of the acute phase, as well as between the end of the acute phase and the 3MFU were calculated. We defined that an increase of the EQ-5D-5L index scores of at least 0.2 between the respective time points denotes an upward trend of the HrQoL and a decrease of at least 0.2 a downward trend. Index scores in between were considered constant. This categorization resulted in nine trend groups.

### Selection of the cohort

**Fig.1:**
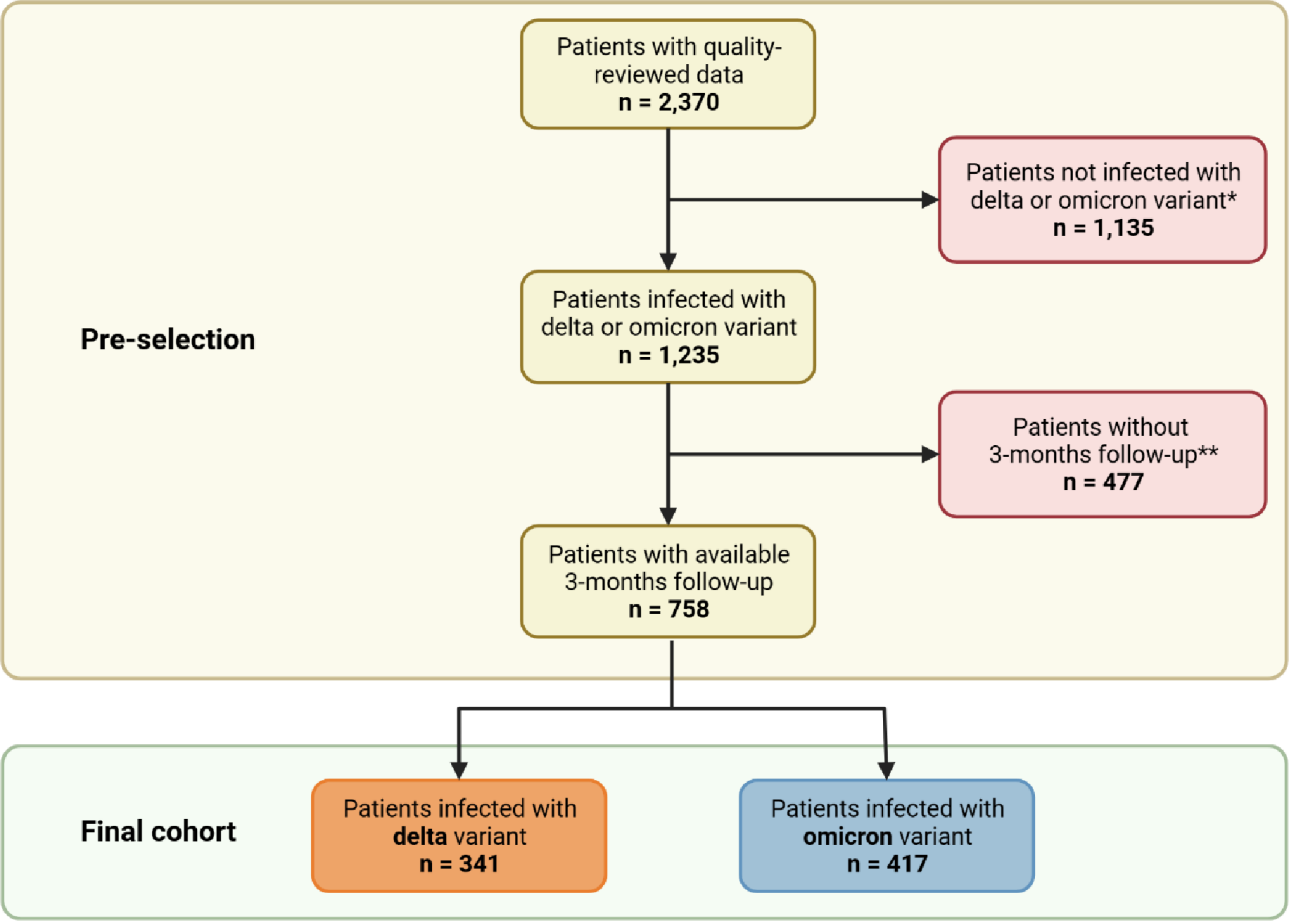
Study flow chart designed by Biorender; *includes (1) patients sequenced for other than delta or omicron variant or patients tested positive (2) before 2021–07-19 or (3) between 2021-12-06 and 2022-01-17 without sequencing for the underlying SARS-CoV-2 variant; **patients either died or were lost to follow-up

For this analysis, we preselected adults that were infected with the SARS-CoV-2 variants delta or omicron and had a quality-reviewed documentation of the 3MFU (Fig. 1). If available, SARS-CoV-2 sequencing results were used to categorize the patients with regard to the SARS-CoV-2 variant of concern. Based on the fact that between July 19, 2021 and December 06, 2021, over 95% of the patients in Germany were infected with the delta variant according to the data of the Robert Koch-Institute (RKI), patients without a SARS-CoV-2 sequencing during that time were assigned to the delta variant [4]. The RKI is the central public health institute in Germany. As of January 17, 2022, more than 95% of SARS-CoV-2 positive patients were infected with the omicron variant in Germany [35]. Therefore, patients without a SARS-CoV-2 sequencing result and a positive SARS-CoV-2 swab since that date were grouped as omicron patients. No distinction was made between the subvariants of omicron. Patients that were primarily infected between December 07, 2021 and January 16, 2022, were excluded if no SARS-CoV-2 sequencing results were available.

### Statistical analysis

The data was processed and analyzed using R (R version 4.1.0 (2021-05-18)) [36]. The data preparation was carried out using the R package epicodr [37].

#### Descriptive statistics

Patient characteristics and underlying symptoms were presented as percentages and absolute numbers for categorical variables, while continuous variables were presented as means (95% confidence intervals = 95% CI) for continuous variables. Age was presented as median (range).

Statistical significance was shown using the Pearson’s chi square test, Mann–Whitney U Test or Student’s t test (two-sided), as appropriate, with p < 0.05 as significance level.

#### Logistic regression models

Logistic regression analyses were performed using maximum likelihood estimation using the R function glm (Package stats version 4.1.0). To examine the risk of acute and PCC-related symptoms according to the underlying variant, we employed multivariable logistic regression analyses, providing adjusted odds ratios (aOR) that elucidate the association between each symptom and the likelihood of the underlying variant. Furthermore, multivariable logistic regression analyses were performed to compare and predict the probability of the occurrence of the PCC-related symptoms pain, dyspnea, and fatigue. We included all baseline characteristics that showed significant levels of p < 0.1 in the univariate analyses between delta and omicron patients as co-variables into these multivariable models (Supplementary Table 1). Further, the SARS-CoV-2 variant itself and the acute symptoms were added as independent variables. The amounts of missing data for each variable are shown in Supplementary Table 2. For the logistic regression analyses, we performed a complete case analysis and excluded those with missing data.

### Literature search on post-COVID-19 condition-related symptoms in delta and omicron patients

A current literature search comparing PCC-related symptoms in delta and omicron patients was performed (criteria mentioned in Supplementary Table 3 are in concordance with a recent systematic literature review [38]). All studies published until November 9, 2023 were considered. The search yielded 354 hits, of which 20 were identified as matching and 18 were relevant to the discussion.

## Results

### Overview of the cohort

In the NAPKON-SUEP, between November 4, 2020 and July 25, 2023, 2,747 SARS-CoV-2 patients were enrolled. By July 26, 2023, 2,370 of these patients across 25 university and seven non-university hospitals as well as 11 local medical practices had a quality-reviewed documentation available. After pre-selection of the cohort, we identified 758 patients who were infected either with the SARS-CoV-2 variant omicron (n = 417) or delta (n = 341) (Fig. 1). In 27.3% (n = 208) of these patients, the SARS-CoV-2 variant was assigned by existing PCR sequencing results. The diagnosis date of the delta patients ranged from April 16, 2021 to January 20, 2022. Omicron patients were initially diagnosed between December 13, 2021 and April 25, 2023. The median age of all patients was 53 years (range 18–93) and 41.2% (n = 312) were female (Table 1). The severity of the acute disease course was determined by the highest value reached according to the WHO Clinical Progression Scale [39]. Most of the patients experienced a moderate disease (50.9%, n = 386), followed by a mild (33.2%, n = 252) and severe (5.9%, n = 45) disease – with significant group differences between delta and omicron patients (p < 0.001). Delta patients showed more severe acute SARS-CoV-2 infections than omicron patients. Furthermore, delta and omicron patients significantly differed in terms of age, body mass index (BMI), ethnicity, vaccination status, and several underlying comorbidities (Table 1).

**Table 1:** Clinical characteristics at baseline of all patients infected with the SARS-CoV-2 variants delta and omicron are presented as *n* (%) for categorical variables, mean (95% confidence interval) for continuous variables, and median (range) for age

	Delta (*n* = 341)	Omicron (*n* = 417)	*p* value	Total cohort (*n* = 758)
Sex			0.079*	
Female	128 (37.5%)	184 (44.1%)		312 (41.2%)
Male	213 (62.5%)	233 (55.9%)		446 (58.8%)
Age in years	49 (18–91)	57 (18–93)	< 0.001**	53 (18–93)
BMI	27.9 (27.1–28.6)	26.2 (25.6–26.8)	< 0.001***	26.9 (26.4–27.4)
Ethnicity			0.015*	
African	7 (2.1%)	3 (0.7%)		10 (1.3%)
Arabic	13 (3.8%)	2 (0.5%)		15 (2.0%)
Asian	2 (0.6%)	1 (0.2%)		3 (0.4%)
Caucasian	296 (86.8%)	372 (89.2%)		668 (88.1%)
Latin American	2 (0.6%)	1 (0.2%)		3 (0.4%)
Others	2 (0.6%)	3 (0.7%)		5 (0.7%)
Smoking status			0.063*	
Active smoker	31 (9.1%)	62 (14.9%)		93 (12.3%)
Former smoker	100 (29.3%)	140 (33.6%)		240 (31.7%)
Nonsmoker	180 (52.8%)	208 (49.9%)		388 (51.2%)
At least one SARS-CoV-2 vaccinations	204 (59.8%)	385 (92.3%)	< 0.001*	589 (77.7%)
Maximum reached WHO Progression Scale value			< 0.001*	
Mild disease	98 (28.7%)	154 (36.9%)		252 (33.2%)
Moderate disease	155 (45.5%)	231 (55.4%)		386 (50.9%)
Severe disease	39 (11.4%)	6 (1.4%)		45 (5.9%)
Comorbidities				
Pulmonary disease	44 (12.9%)	83 (19.9%)	0.015*	127 (16.8%)
Cardiovascular disease	130 (38.1%)	195 (46.8%)	0.024*	325 (42.9%)
Hematological and/or oncological disease	52 (15.2%)	99 (23.7%)	0.006*	151 (19.9%)
Liver disease	18 (5.3%)	27 (6.5%)	0.594*	45 (5.9%)
Kidney disease	38 (11.1%)	43 (10.3%)	0.812*	81 (10.7%)
Neurological disease	40 (11.7%)	71 (17.0%)	0.056*	111 (14.6%)
Diabetes mellitus I or II	46 (13.5%)	54 (12.9%)	0.895*	100 (13.2%)

* = Pearsons chi square test; ** = Mann Whitney *U* test. *** = Student’s *t* test. *p* < 0.05 = significant (in bold). *BMI *Body mass index

“At least one SARS-CoV-2 vaccination” is related to the time before the COVID-19 infection

### Symptoms in the acute phase of SARS-CoV-2 infection

**Fig.2:**
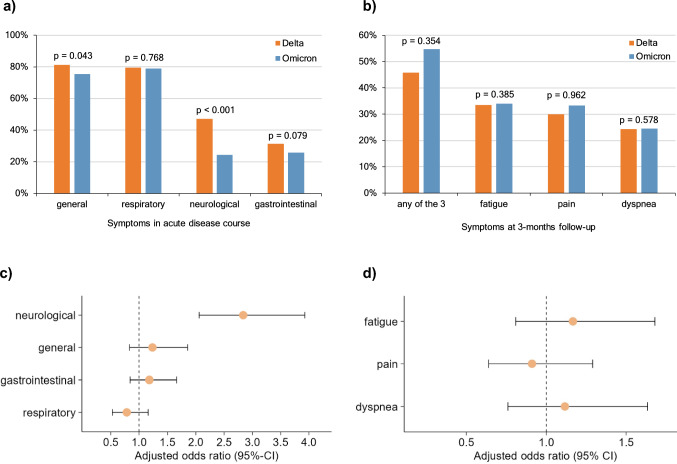
Description of acute and post-COVID-19-condition (PCC)-related symptoms in patients infected with the SARS-CoV-2 variants delta (*n* = 341) and omicron (*n* = 417). The acute symptoms were categorized into general, respiratory, neurological, and gastrointestinal symptoms. Patient Reported Outcome Measures (PROMs) were used to ask the patients for fatigue, pain, and dyspnea at the 3-months follow-up (3MFU). In addition, the number of patients who had at least one of the three PCC-related symptoms was detected (any of the 3). The bar graphs show the symptom prevalence in the acute disease course (**a**) and at the 3MFU (**b**). Significance levels between delta and omicron patients were computed using Pearsons chi square test, *p* < 0.05 = significant. The associations between the acute symptoms (*n* = 735 after deletion of patients with missing data) (**c**) and the PCC-related symptoms at the 3MFU (*n* = 691 after deletion of patients with missing data) (**d**) and the underlying SARS-CoV-2 variant were calculated with a multivariable logistic regression model. Adjusted odds ratios and 95% confidence intervals (95%-CI) were determined to compare delta with omicron patients

In the acute phase of COVID-19 disease, general and respiratory symptoms appeared frequently in all patients (78.0%, n = 591 and 79.2%, n = 600, Fig. 2a). Compared with omicron patients, delta patients had significantly more general (75.3%, n = 314 vs. 81.2%, n = 277; p = 0.043) and neurological (24.2%, n = 101 vs. 46.9%, n = 160; p < 0.001) symptoms. At the same time, the frequency of respiratory (79.5%, n = 271, vs. 78.9%, n = 329; p = 0.768) and gastrointestinal symptoms (31.4%, n = 107 vs. 25.7%, n = 107; p = 0.079) did not differ significantly between the delta and omicron patients, respectively. Regarding the multivariable regression, we found that delta patients presented more likely with neurological symptoms in the acute phase than omicron patients (aOR 2.84, 95%-CI 2.06–3.93, p < 0.001, Fig. 2c).

### Prevalence of post-COVID-19 condition-related symptoms at the 3-months follow-up

Three months after primary infection, patients were asked for pain, dyspnea and fatigue. Fifty-one percent of all patients reported at least one of the three symptoms (Fig. 2b). The prevalence did not differ significantly between delta and omicron patients (45.7%, n = 156 vs. 54.7%, n = 228; p = 0.354). Among all patients, fatigue was the most frequently reported symptom (33.8%, n = 256), followed by pain (31.8%, n = 241) and dyspnea (24.4%, n = 185). Similarly, the multivariable logistic regression did not show a significantly different risk for the occurrence of PCC-related symptoms according to the underlying SARS-CoV-2 variant (Fig. 2d). Patients treated as outpatients during the acute course had fewer PCC-related symptoms than hospitalized patients, although no significant differences were observed between the underlying SARS-CoV-2 variants (Supplementary Fig. 1 a-b). Patients aged 60 or older reported more PCC-related symptoms than patients under 60 years old. Among younger patients (18–59 years), significantly more delta than omicron patients had dyspnea at the 3MFU (Supplementary Fig. 1 c–d).

### Associations between symptoms in the acute phase and the presence of post-COVID-19 condition-related symptoms at the 3-months follow-up

Results of multivariable regression models on the risk for PCC-related symptoms are demonstrated in Table 2. Patients with acute gastrointestinal (aOR 2.23, 95% CI 1.46–3.43, p < 0.001) and neurological (aOR 1.58, 95% CI 1.03–2.42, p = 0.035) symptoms had a higher risk to experience pain as PCC-related symptom. In contrast, acute general and respiratory symptoms did not affect the risk for pain at the 3MFU. The risk for PCC-related pain and dyspnea was further increased by a higher body mass index, respectively (aOR 1.08, 95% CI 1.04–1.12, p < 0.001; aOR 1.04, 95% CI 1.00–1.08, p = 0.044).

**Table 2:** Associations between symptoms in the acute phase and the presence of pain (*n* = 537 patients after deletion of patients with missing data), dyspnea (*n* = 536 patients after deletion of patients with missing data), and fatigue (*n* = 532 patients after deletion of patients with missing data) at the 3-months follow-up

	Pain		Dyspnea		Fatigue	
	aOR (95% CI)	*p* value	aOR (95%-CI)	*p* value	aOR (95%-CI)	*p* value
SARS-CoV-2 variant of concern						
Omicron	ref	ref	ref	ref	ref	ref
Delta	0.85 (0.53–1.33)	0.468	0.94 (0.56–1.56)	0.821	1.08 (0.69–1.70)	0.734
Symptoms in the acute phase						
General	1.61 (0.96–2.77)	0.078	0.67 (0.38–1.20)	0.175	1.26 (0.75–2.14)	0.387
Respiratory	1.13 (0.69–1.88)	0.623	2.47 (1.35–4.68)	**0.004**	2.38 (1.41–4.13)	**0.001**
Gastrointestinal	2.23 (1.46–3.43)	**< 0.001**	1.73 (1.07–2.78)	**0.024**	1.84 (1.20–2.82)	**0.005**
Neurological	1.58 (1.03–2.42)	**0.035**	1.44 (0.89–2.33)	0.132	1.29 (0.84–1.97)	0.240
Baseline characteristics						
Male	0.74 (0.50–1.11)	0.143	0.96 (0.61–1.53)	0.879	0.82 (0.55–1.22)	0.328
Age	1.01 (0.99–1.02)	0.213	1.01 (0.99–1.03)	0.135	0.99 (0.98–1.01)	0.436
Body mass index	1.08 (1.04–1.12)	**< 0.001**	1.04 (1.00–1.08)	**0.044**	1.02 (0.99–1.06)	0.206
Active smoker	1.23 (0.69–2.16)	0.474	1.52 (0.79–2.87)	0.197	1.30 (0.74–2.27)	0.362
At least one SARS-CoV-2 vaccination	1.27 (0.74–2.21)	0.382	1.09 (0.60–2.00)	0.787	1.63 (0.94–2.86)	0.084
WHO Progression Scale phase	1.62 (1.10–2.40)	**0.015**	2.35 (1.52–3.69)	**< 0.001**	1.81 (1.24–2.68)	**0.003**
Pulmonary disease	1.17 (0.70–1.92)	0.545	3.03 (1.80–5.10)	**< 0.001**	1.59 (0.97–2.63)	0.067
Cardiovascular disease	0.94 (0.58–1.52)	0.803	1.46 (0.86–2.48)	0.160	1.00 (0.62–1.62)	0.993
Hematological/oncological disease	1.07 (0.65–1.76)	0.780	0.91 (0.52–1.55)	0.720	1.28 (0.78–2.10)	0.322
Neurological disease	1.58 (0.94–2.66)	0.083	0.94 (0.53–1.66)	0.843	1.76 (1.04–2.98)	**0.035**

Results from multivariable logistic regression models displayed with adjusted odds ratios (aOR) and 95% confidence intervals (95% CI). Baseline characteristics with significant differences between delta and omicron patients in the univariate regression model were included as co-factors. In binary variables, no reference was indicated. A *p* value < 0.05 indicates a significant difference (in bold)

The occurrence of respiratory and gastrointestinal symptoms in the acute phase of COVID-19 disease resulted in a higher risk of dyspnea (aOR 2.47, 95% CI 1.35–4.68, p = 0.004; aOR 1.73, 95% CI 1.07–2.78, p = 0.024) and fatigue (aOR 2.38, 95% CI 1.41–4.13, p = 0.001; aOR 1.84, 95% CI 1.20–2.82, p = 0.005) at the 3MFU, respectively. Patients with pulmonary comorbidities demonstrated a significantly higher risk for PCC-related dyspnea (aOR 3.03, 95% CI 1.80–5.10, p < 0.001) while those with neurological comorbidities had a significantly higher risk for PCC-related fatigue (aOR 1.76, 95% CI 1.04–2.98, p = 0.035). A higher value on the WHO Progression Scale during the acute phase significantly raised the risk for pain, dyspnea, and fatigue at the 3MFU. The presence of the delta or omicron variant did not have a significant influence on the prevalence of PCC-related symptoms.

### Evaluating the severity of post-COVID-19 conditions

HrQoL indicator analysis is demonstrated in Table 3. With 0.75 (95% CI 0.73–0.78), the EQ-5D-5L index score was lowest at the beginning of the COVID-19 disease, indicating the greatest impairment for the patient. On average, delta and omicron patients showed signs of recovery until the end of the acute phase with a mean EQ-5D-5L index score of 0.85 (delta: 95% CI 0.82–0.88; omicron: 95% CI 0.82–0.87). With regard to the EQ-VAS, both groups improved steadily from the onset of the COVID-19 disease to the 3MFU.

**Table 3:** Differences in EQ-5D-5L index scores between delta (*n* = 341) and omicron (*n* = 417) patients at three different visit time points

	Delta	Omicron	*p* value 1	*p* value 2
	Mean (95% CI)	Mean (95% CI)		
EQ-5D-5L index scores				
At baseline	0.74 (0.69–0.78)	0.76 (0.73–0.79)	0.360	0.338
At end of acute phase	0.85 (0.82–0.88)	0.85 (0.82–0.87)	0.513	0.988
At 3-months follow-up	0.86 (0.84–0.89)	0.84 (0.81–0.86)	0.452	0.200
EQ-VAS scores				
At baseline	59.6 (56.8–62.4)	62.4 (60.0–64.7)	0.075	0.136
At end of acute phase	69.8 (67.1–72.6)	72.6 (70.4–74.8)	0.122	0.122
At 3-months follow-up	78.1 (76.0–80.1)	75.8 (73.6–77.9)	0.482	0.135

Since we do not assume a normal distribution of the index scores, significance levels between delta and omicron patients were computed using Mann Whitney *U* test (*p* value 1) and Student’s *t* test (*p* value 2) with *p* < 0.05 = significant. The results were similar and showed no significant values respectively. *95% CI* 95% confidence interval

In the next step, trend groups were calculated to describe the EQ-5D-5L index score changes between the baseline and the end of the acute phase, as well as the end of the acute phase and the 3MFU (Table 4). Most patients (69.0%, n = 348) never showed a decline of HrQoL after baseline and remained in a high HrQoL group from baseline to 3MFU (trend group 1). The second most frequent trend group (trend group 2) observed were patients who—after an initially impaired HrQoL (mean EQ-5D-5L index score 0.36, 95% CI 0.29–0.43)—improved by the end of the acute phase (mean EQ-5D-5L index score 0.86, 95% CI 0.82–0.90), followed by a consistently high HrQoL at the 3MFU (mean EQ-5D-5L index score 0.88, 95% CI 0.84–0.93).

**Table 4:**
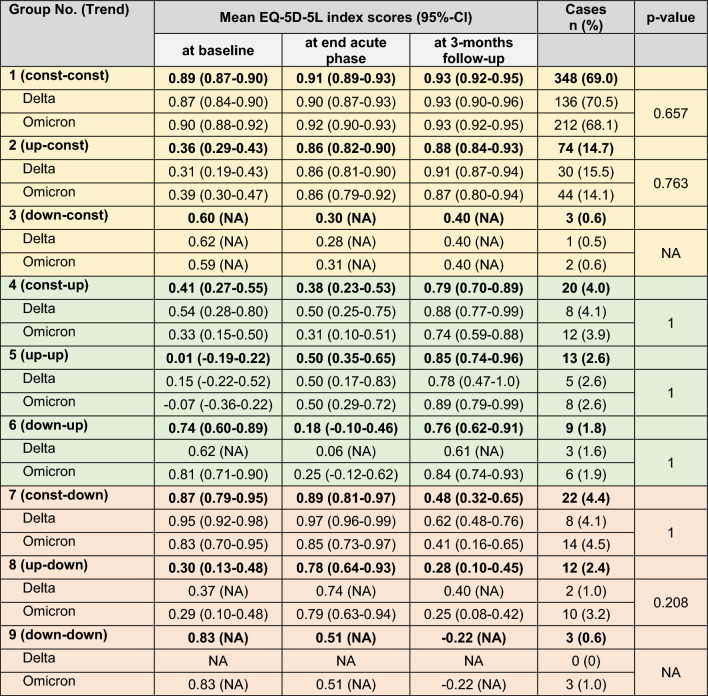
The EQ-5D-5L index score changes between baseline and end of acute phase, as well as end of acute phase and 3-months follow-up (3MFU) were categorized into trend groups

With regard to each single patient, an improvement of at least 0.2 describes an upward trend (“up”), a deterioration of at least 0.2 a downward trend (“down”), in between the trends are considered constant (“const”). The first trend given describes the trend between the baseline visit and the end of the acute phase, the second between the end of the acute phase and the 3MFU. Means of EQ-5D-5L index scores are shown for the respective trend groups, subdivided into all (*n* = 504), delta (*n* = 193) and omicron (*n* = 311) patients. Only patients without missing data at each time point (baseline, end acute phase, 3MFU) were considered. Significance levels between delta and omicron cases were computed using Pearsons chi square test, *p* < 0.05 = significant. *95% CI* 95% confidence interval. If the case number was less than 4, no confidence intervals were calculated (indicated as NA)

For 37 patients (7.3%), the HrQoL deteriorated substantially from the acute phase to the 3MFU (trend groups 7, 8, 9). Compared to the patients in trend groups one to six, these trend groups were characterized by significantly older patients in median (62 years, range 20–85 vs. 50 years, range 18–92; p = 0.008), but a similar amount of omicron patients (73%, n = 27/37 vs. 61%, n = 284/467; p = 0.197). Furthermore, patients of trend groups seven to nine suffered from significantly more PCC-related pain (68%, n = 25/37 vs. 27%, n = 126/467; p < 0.001), fatigue (54%, n = 20/37 vs. 30%, n = 139/467; p = 0.006), and dyspnea (43%, n = 16/37vs. 19%, n = 87/467; p = 0.001) at the 3MFU than patients of the other trend groups.

## Discussion

In our study, we analyzed potential differences in the occurrence of PCC-related symptoms between patients infected with the delta and the omicron variant. As this was the main focus of our analysis, we just described the acute symptoms of the pre-selected cohort of patients with existing 3MFU. In the acute phase of the COVID-19 disease, delta patients in our cohort suffered from more symptoms than omicron patients, especially regarding general and neurological acute symptoms. We found that occurrence of acute respiratory, neurological, and gastrointestinal symptoms was predictive for PCC-related finding at 3MFU. However, the underlying variant responsible for acute symptoms had no significant effect on the occurrence of PCC. Our results of the symptoms in the acute phase of COVID-19 disease match with previous studies on the differences between the SARS-CoV-2 variants delta and omicron [16, 21]. For example, a prospective observational study from the United Kingdom showed that the neurological symptoms loss of smell and loss of taste were less common in omicron than in delta patients [16]. It must be noted that in our analysis, patients with severe illnesses in the acute phase and subsequent deaths were not considered as only patients with existing 3MFU were included.

Preliminary studies on earlier SARS-CoV-2 variants showed that differences in the prevalence of PCC-related symptoms may be present depending on the virus variant: PCC-related symptoms of the group of neurological and cognitive/emotional categories appearing four to 12 weeks after primary infection were found to differ in SARS-CoV-2 infected patients, if the wild-type variant compared to the alpha variant was prevalent [40]. Patients infected in the first wave of COVID-19 in early 2020 (historical cohort) experienced more PCC-related dyspnea than patients infected with the alpha or delta variant six months after SARS-CoV-2 infection. At the same time, the prevalence of PCC-related fatigue was not affected by these variants [41]. Our analysis confirmed that fatigue at the 3MFU was equally frequent in delta and omicron patients. However, this was also the case for dyspnea, which distinguished it from the mentioned studies comparing earlier SARS-CoV-2 variants underlining that infections with the delta and omicron variant had similar effects on PCC. Only among the younger patients (18-59 years) we observed significantly more dyspnea at the 3MFU in delta compared to omicron patients.

In addition to fatigue as the most common PCC-related symptom, pain is frequently reported [38]. A comparison between a historical, alpha and delta cohort revealed a similar prevalence of de novo musculoskeletal pain six months after primary infection [42]. In our analysis, we were able to demonstrate an equally frequent occurrence of PCC-related pain comparing delta and omicron patients three months after infection.

Literature reveals that the prevalence of PCC-related symptoms between patients infected with the delta and omicron variant differed between published studies: (1) The first part of the studies showed fewer PCC-related symptoms in omicron patients compared to those infected with other SARS-CoV-2 variants [43–46]. For example, one of the first comparisons in delta and omicron patients showed that overall, omicron patients had a decreased risk of PCC-related symptoms, compared to delta patients with no distinction in the type of symptoms [47, 48]. This was also shown in a Spanish study in which omicron patients had significantly fewer PCC-related symptoms than alpha or delta patients at least 12 weeks after primary infection [49] . In a subgroup of hematological patients, the risk of PCC-related symptoms decreased from a historical cohort to alpha, delta and omicron patients [50]. In contrast to these studies, we found no significant differences in the prevalence of PCC between delta and omicron patients in our cohort at the 3MFU. This could be explained by the fact that part of the mentioned studies represented specific subpopulations whereas in our study, a broad group of adult patients (18 to 93 years with different comorbidities) was investigated. In addition, the time of PCC occurrence differed between the studies which made comparability impossible. We performed subgroup analyses with regard to the hospitalization status and age. There were no differences in the prevalence of PCC-related symptoms between delta and omicron patients in the subgroups of outpatients, hospitalized and elderly (≥ 60 years) patients. In contrast, among younger patients (18-59 years), significantly more delta than omicron patients reported dyspnea at the 3MFU. (2) The second part of the studies reported no differences between the prevalence of PCC of omicron patients compared to patients infected with other variants. For example, a pooled data analysis of population-based cohorts in Switzerland could not detect any differences in patterns of the PCC-related symptom clusters (systemic, neurocognitive, cardiorespiratory, and musculoskeletal symptom clusters) across patients infected with wildtype SARS-CoV-2 as well as delta and omicron variants [51] . A systematic literature review up to July 2022 revealed that PCC-related symptoms did not differ depending on the various virus variants [52]. This was confirmed by data from prospective studies in Norway [53], South Africa [54], and the United States [55], as well as by a retrospective study from Hungary [56]. We found one study that examined PCC-related symptoms 1.5 years after the primary infection [57]. The majority of these patients showed no improvement in the severity of PCC, independent of the SARS-CoV-2 variants (wild-type, alpha, delta, and omicron). According to the results of the studies mentioned under (2), we could not detect any significant differences in PCC-related symptoms between delta and omicron patients. Our results stand out from the mentioned studies by the fact that our data was collected quality controlled from a prospective multicenter cohort conducted in entire Germany.

In an attempt to better separate general health conditions unrelated to the SARS-CoV-2 infection from attributable PCC-related symptoms, we took a unique approach of grouping related trends in HrQoL from the acute phase of COVID-19 disease over the recovery up to the 3MFU, using EQ-5D-5L index scores. We were able to show that over two thirds of patients did not experience any restrictions in their HrQoL due to or after the SARS-CoV-2 infection three months after primary infection compared to the mean EQ-5D-5L index score of 0.88 of a representative German adult population [34, 58] . Nevertheless, at the same time, around half of the patients reported to experience at least one of the PCC-related symptoms fatigue, pain or dyspnea. This finding suggests that despite perceiving symptoms, an impairment in HrQoL is not always present, but may also hint at pre-existing conditions that patients have become accustomed to and are not perceived as limiting HrQoL. The trend groups also revealed that approximately 7% (n = 37) of the patients experienced a deterioration of their HrQoL after the acute illness, with slightly more omicron than delta patients affected.

Despite highest effort in setting up and conducting the cohort [31, 32], our study is limited by missing data, mostly based on delayed introduction of some data items relevant for this analysis over the course of the recruitment. Furthermore, no subdivision of the results into the different omicron subtypes was possible, as variant sequencing was no longer conducted in Germany at the time. For this reason, we partly had to base the assumption of the infecting variant on the distributions in Germany according to the weekly reports of the RKI. Apart from the SARS-CoV-2 vaccination status, we had no information on secondary SARS-CoV-2 infections or on the COVID-19 serology prior to the documented infection.

## Conclusion

Our results from a large prospective multicenter and cross-sectoral cohort showed that patients infected with the SARS-CoV-2 variant omicron had fewer symptoms in the acute phase of COVID-19 disease than delta patients. However, after controlling for established risk-factors, both variants lead to equally frequent PCC-related symptoms. We were able to demonstrate that despite the change in SARS-CoV-2 variants and thus reduced acute disease severity, PCC-related symptoms were an equally common challenge for both variants. Furthermore, we found that a reported PCC-related symptom, detected in 51% of all patients, did not necessarily indicate impairment in everyday life as over two thirds of all patients did not experience any restrictions in their HrQoL. It was generally difficult to assess the severity of individual PCC-related symptoms, which may also be an indicator of over-diagnosis of PCC based on established criteria.

## Supplementary Information

Below is the link to the electronic supplementary material.

Supplementary file1 (PDF 152 KB)

## Data Availability

The data that supports the findings of this study is available from the authors but restrictions apply to the availability of this data, which was used in compliance with the NAPKON Usage and Publication Regulations for the current study, and so is not publicly available. Data is, however, available from the authors upon reasonable request and with permission from the NAPKON Use and Access Committee (https://napkon.de/use-and-access/).

## Abbreviations

aOR: Adjusted odds ratio
CI: Confidence interval
COVID-19: Coronavirus Disease 2019
EQ VAS: EQ visual analogue scale
HrQoL: Health-related quality of life
NAPKON: German National Pandemic Cohort Network
NUKLEUS: NUM Clinical Epidemiology and Study Platform
NUM: Network University Medicine
PCC: Post-COVID-19 condition
PROMs: Patient Reported Outcome Measures
RKI: Robert Koch-Institute
SARS-CoV-2: Severe Acute Respiratory Syndrome Coronavirus 2
SUEP: Cross-Sectoral Platform
WHO: World Health Organization
3MFU: 3-Months follow-up
