# Supplementary material for: Comparison of post-COVID-19 symptoms in patients infected with the SARS-CoV-2 variants delta and omicron—results of the Cross-Sectoral Platform of the German National Pandemic Cohort Network (NAPKON-SUEP)
Source: Infection. 2024 May 3;52(6):2253–67. doi: 10.1007/s15010-024-02270-5 (PMC11621170; doi:10.1007/s15010-024-02270-5)
Supplement: Supplementary file 1 — Supplementary file1 (PDF 152 KB) [file 15010_2024_2270_MOESM1_ESM.pdf]

Supplementary

Supplementary Table 1 Differences in baseline characteristics in patients infected with the SARS-CoV-2 variants omicron vs. delta. Results from univariate logistic regression models displayed with adjusted odds ratios (aOR) and 95% confidence intervals (95%-CI). If the p-value was < 0.1, the respective baseline characteristic was included as co-factor in the multivariable logistic regression models. Due to the very low case numbers in the ethnicity subgroups, the variable was only categorized as Caucasian and non-Caucasian.

Baseline characteristics	aOR (95%-CI)	p-value
Male	1.31 (0.98-1.76)	**0.067**
Age	0.99 (0.98-0.99)	**< 0.001**
Body Mass Index	1.05 (1.02-1.08)	**< 0.001**
Caucasian	0.80 (0.51-1.24)	0.309
Active smoker	0.57 (0.36-0.90)	**0.017**
Former smoker	0.82 (0.60-1.12)	0.211
Nonsmoker	1.12 (0.84-1.50)	0.426
At least one SARS-CoV-2 vaccination	0.12 (0.08-0.19)	**< 0.001**
WHO Progression Scale phase	1.69 (1.30-2.20)	**< 0.001**
Pulmonary disease	0.60 (0.40-0.89)	**0.012**
Cardiovascular disease	0.71 (0.53-0.95)	**0.020**
Hematological/ oncological disease	0.58 (0.40-0.84)	**0.005**
Liver disease	0.81 (0.43-1.48)	0.492
Kidney disease	1.09 (0.68-1.73)	0.722
Neurological disease	0.65 (0.43-0.99)	**0.045**
Diabetes mellitus I and II	1.05 (0.69-1.61)	0.810

Supplementary Table 2 Missing analysis: All variables for regression analysis containing missing values were analyzed with regard to missing rates and p-value of the test on missing completely at random (MCAR). P < 0.05 = significant, meaning MCAR-hypothesis is rejected.

Parameter	Missing values % (no.)	Test on MCAR-hypothesis (p-value)
Body Mass Index	14.4 (109/758)	0.623
Smoking status	4.9 (37/758)	0.144
WHO Progression Scale phase	9.9 (75/758)	0.911
Pulmonary disease	0.9 (7/758)	0.379
Cardiovascular disease	0.3 (2/758)	0.239
Hematological/ oncological disease	2.4 (18/758)	0.118
Neurological disease	2.5 (19/758)	0.408
General symptoms acute phase	0.9 (7/758)	0.678
Respiratory symptoms acute phase	0.8 (6/758)	0.110
Gastrointestinal symptoms acute phase	1.2 (9/758)	0.969
Neurological symptoms acute phase	1.8 (14/758)	0.823
Pain at 3-months follow-up	6.6 (50/758)	0.365
Dyspnea at 3-months follow-up	6.7 (51/758)	0.600
Fatigue at 3-months follow-up	7.8 (59/758)	0.145
Any symptom at 3-months follow-up	7.1 (54/758)	0.194

Supplementary Table 3 Literature search for studies describing the prevalence of post-COVID-19 condition-related symptoms of omicron patients compared to delta patients.

Search number	PubMed Search Formula
**#1**	"post acute covid 19 syndrome" [All Fields] OR “long-COVID” [All Fields] OR “long-COVID symptoms” [All Fields] OR “long hauler” [All Fields] OR “post-COVID-19” [All Fields] OR “post-acute COVID-19 symptoms” [All Fields] OR “COVID-19 sequelae”
**#2**	“delta” [All Fields] OR “B.1.617.2” [All Fields] AND “omicron” [All Fields] OR “B.1.1.529”
**#3**	#1 AND #2

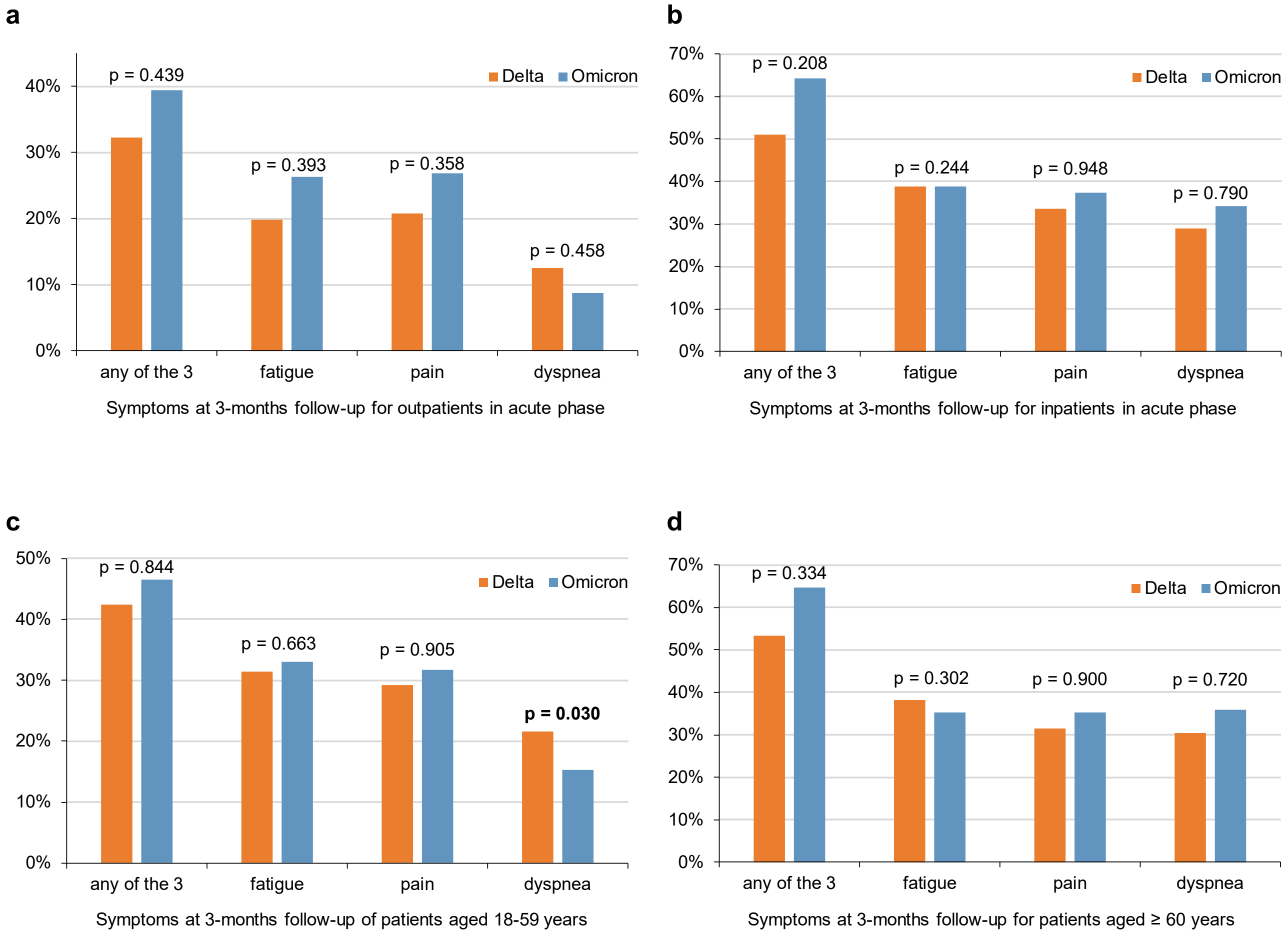

Supplementary Figure 1 Description of post-COVID-19-condition (PCC)-related symptoms in patients infected with the SARS-CoV-2 variants delta and omicron depending on the hospitalization status in the acute phase as well as the age. Screening questions were used to ask the patients for fatigue, pain, and dyspnea at the 3-months follow-up (3MFU). In addition, the number of patients who had at least one of the three PCC-related symptoms was detected (any of the 3). The bar graphs show the PCC-related symptom prevalence for (**a**) ambulatory (delta n = 96; omicron n = 160) and (**b**) hospitalized (delta n = 245; omicron n = 257) patients with regard to the acute phase of COVID-19 disease, as well as for patients aged (**c**) 18-59 years (delta n = 236; omicron n = 230) and (**d**) ≥ 60 years (delta n = 105; omicron n = 187). Significance levels between delta and omicron patients were calculated using the Person's chi square test, with $p < 0.05$ considered significant.
